# Supplementary material for: Stress hyperglycemia ratio predicts adverse outcomes in emergency department patients with upper gastrointestinal bleeding
Source: PLoS One. 2026 Jan 23;21(1):e0340450. doi: 10.1371/journal.pone.0340450 (PMC12829839; doi:10.1371/journal.pone.0340450)
Supplement: S1 Table — Unadjusted and adjusted odds ratios (ORs) with 95% confidence intervals (CIs) were calculated using logistic regression analyses. Multivariable models were adjusted for age, sex, hepatic disease, and chronic heart failure. ICU, intensive care unit; AKI, acute kidney injury; AMI, acute myocardial infarction; ARF, acute respiratory failure. *P < 0.05. (DOCX) [file pone.0340450.s001.docx]

**Table S1.** **Association between stress hyperglycemia ratio and adverse outcomes in patients with and without diabetes mellitus.**

**A. Patients with diabetes mellitus**

| Outcome | Unadjusted OR  (95% CI) | *P*-value | Adjusted OR  (95% CI) | *P*-value |
| --- | --- | --- | --- | --- |
| Blood transfusion | 4.15 (1.57-11.00) | < 0.001* | 6.02 (2.00-18.13) | < 0.001* |
| ICU admission | 2.33 (1.33-4.08) | < 0.001* | 2.18 (1.25-3.80) | < 0.001* |
| Rebleeding | 1.27 (0.35-4.59) | 0.72 | 1.31 (0.28-6.23) | 0.73 |
| Intervention | 1.20 (0.77-1.85) | 0.42 | 1.03 (0.65-1.64) | 0.89 |
| Mortality | 0.16 (0.03-1.79) | 0.16 | 0.33 (0.05-2.09) | 0.24 |
| AKI | 1.66 (1.07-2.56) | 0.02* | 1.89 (1.19-3.01) | < 0.001* |
| AMI | 1.16 (0.38-3.57) | 0.80 | 1.55 (0.51-4.67) | 0.44 |
| ARF | 1.08 (0.46-2.51) | 0.87 | 1.11 (0.45-2.73) | 0.83 |

**B. Patients without diabetes mellitus**

| Outcome | Unadjusted OR  (95% CI) | *P*-value | Adjusted OR  (95% CI) | *P*-value |
| --- | --- | --- | --- | --- |
| Blood transfusion | 7.77 (1.69-35.83) | < 0.001* | 8.13 (1.53-43.32) | 0.01* |
| ICU admission | 2.14 (1.16-3.95) | 0.02* | 1.87 (0.96-3.66) | 0.07 |
| Rebleeding | 1.85 (1.02-3.36) | 0.04* | 2.61 (1.25-5.48) | 0.01* |
| Intervention | 1.19 (0.76-1.86) | 0.45 | 1.23 (0.70-1.81) | 0.62 |
| Mortality | 0.60 (0.07-5.38) | 0.65 | 0.16 (0.01-3.38) | 0.24 |
| AKI | 1.51 (0.92-2.49) | 0.11 | 1.59 (0.90-2.80) | 0.11 |
| AMI | 0.87 (0.27-2.80) | 0.81 | 1.06 (0.25-4.43) | 0.94 |
| ARF | 3.17 (1.51-6.64) | < 0.001* | 3.62 (1.45-9.07) | < 0.001* |

Unadjusted and adjusted odds ratios (ORs) with 95% confidence intervals (CIs) were calculated using logistic regression analyses. Multivariable models were adjusted for age, sex, hepatic disease, and chronic heart failure.

ICU, intensive care unit; AKI, acute kidney injury; AMI, acute myocardial infarction; ARF, acute respiratory failure.

*P < 0.05.
