# Supplementary material for: Stress hyperglycemia ratio predicts adverse outcomes in emergency department patients with upper gastrointestinal bleeding
Source: PLoS One. 2026 Jan 23;21(1):e0340450. doi: 10.1371/journal.pone.0340450 (PMC12829839; doi:10.1371/journal.pone.0340450)
Supplement: Attachment — Submitted filename: Response_to_Reviewers.docx [file pone.0340450.s003.docx]

Dear Academic Editor and Reviewer,

We sincerely appreciate the constructive comments provided. We have revised the manuscript accordingly.

Below, we provide a point-by-point response to all comments. All changes refer to the revised manuscript with tracked changes.

=============================================================

**Response to Academic Editor**

(Note: The numbering of comments follows the Academic Editor’s original list, in which comment #3 was not present.)

**Editor Comment 1:**

**“typing errors”**

Response:

We thank the Academic Editor for noting this issue. We have carefully reviewed the entire manuscript and corrected all typographical, grammatical, and formatting errors to improve clarity and readability.

**Editor Comment 2:**

**“The authors adjust the SHR > 1.9; add the sensitivity and specificity for this cut-off value because we can’t calculate Youden’s index without sensitivity and specificity.”**

Response:

We appreciate the Editor’s comment. The optimal cut-off derived from the ROC analysis for predicting ICU admission was **1.898** based on the Youden index. For clarity and clinical applicability, we rounded this value to **1.9**, which did not materially change the diagnostic performance. At this cut-off, sensitivity was **46.4%** and specificity was **81.0%**, and these values have now been added to the Materials and Methods section.

Changes in Manuscript:

Sensitivity and specificity values corresponding to the SHR cutoff of 1.9 were added to the Statistical Analysis section of the Materials and Methods.

**Editor Comment 4:**

**“Add reference for the eAG formula.”**

Response:

Thank you for the comment. We have added the original ADAG study by Nathan et al. (Diabetes Care, 2008) as the reference for the eAG formula[1].

Changes in Manuscript:

A reference supporting the eAG formula was added to the Measurements subsection of the Materials and Methods.

**Editor Comment 5:**

**“How the author deals with missing data. Flow chart is necessary to outline the process of methodology.”**

Response:

Thank you for this important comment. When retrieving cases from the hospital data center, only patients who had both an upper endoscopy performed during the index visit and an available HbA1c measurement were provided to the research team. Therefore, cases lacking HbA1c data or endoscopic evaluation were excluded at the data extraction stage, and all variables required for calculating the stress hyperglycemia ratio were complete. No imputation was performed.

Although blood urea nitrogen (BUN) had partial missing values, it was not included in the calculation of the stress hyperglycemia ratio or in the primary predictive models and therefore did not affect the study conclusions.

Changes in Manuscript:

The Methods section was updated to describe the handling of missing data, and a study flowchart illustrating patient selection was added as Figure 1.

**Editor Comment 6:**

**“The tables showed that the continuous data are not normally distributed. Therefore the results mean ± SD, as well as the analysis will bias the conclusion.”**

Response:

Thank you for this important comment. We re-evaluated the distribution of all continuous variables using the Shapiro–Wilk test. As shown in the updated analysis, most continuous variables—including admission glucose, HbA1c, glycemic gap, SHR, hemoglobin, and hospital stay—were non-normally distributed (Shapiro–Wilk p < 0.05). Only age demonstrated approximate normality.

To address this issue and avoid biased interpretation, we have revised Table 1 and now present all continuous variables as median with interquartile range instead of mean ± SD. Correspondingly, the Statistical Analysis section has been updated to reflect the use of Shapiro–Wilk testing and the non-normal distribution of these variables. These revisions ensure that data presentation is statistically appropriate and consistent with the underlying distribution.

Changes in Manuscript:

Revisions were made to the Methods (Statistical Analysis section), the Results (Baseline characteristics), and Table 1.

**Editor Comment 7:**

**“Tables are not clear (the title, and footnotes).”**

Response:

Thank you for the comment. We have revised all table titles and footnotes to improve clarity and consistency. Abbreviations are now fully defined, statistical methods are clearly stated, and redundant wording has been removed. We believe the tables are now clearer and easier to interpret.

Changes in Manuscript:

We revised the titles and footnotes of Tables 1–4 and Supplementary Table S1, clarified statistical methods, fully defined abbreviations, and standardized formatting across all tables.

**Editor Comment 8:**

**“Improve the production of Figures.”**

Response:

Thank you for this comment. All figures were carefully revised and reformatted to ensure clear layout, consistent typography, and appropriate visual contrast. All figures were prepared and provided as high-resolution TIFF files in accordance with PLOS ONE figure requirements. We believe that the revised figures substantially improve clarity and overall presentation.

Changes in Manuscript:

All figures were revised and reformatted to improve clarity, consistency, and visual quality.

**Editor Comment 9:**

**“Update the references as >40% are old references.”**

Response:

Thank you for this important comment. We have carefully reviewed and updated the reference list to address this concern. Older references were replaced with more recent and relevant studies wherever appropriate, particularly in sections discussing stress hyperglycemia, glycemic gap, and clinical outcomes. Foundational and landmark references were retained only when necessary to support established concepts, risk scores, or pathophysiological mechanisms. As a result, the majority of references now consist of publications from the past decade, and the overall reference list has been substantially updated to reflect current evidence.

=============================================================

**Response to Reviewer #1**

**Reviewer Comment 1:**

**“It would be helpful for the reader to speculate on potential mechanistic associations between transfusion and stress hyperglycemia, and similarly between rebleeding and stress hyperglycemia; while this is not the goal of the study, it would help place the interesting findings in context. ”**

Response:

We sincerely thank the reviewer for this insightful suggestion. In accordance with the comment, we have added mechanistic explanations to clarify the potential biological and physiological pathways linking stress hyperglycemia with both transfusion requirements and rebleeding risk.

For transfusion, we now describe that acute hemorrhage triggers sympathetic and hypothalamic–pituitary–adrenal (HPA) axis activation, leading to catecholamine- and cortisol-mediated hyperglycemia, and how this stress response reflects the severity of bleeding.

For rebleeding, we further elaborate on two complementary pathways: (1) SHR as a marker of initial bleeding severity—a well-recognized determinant of rebleeding in UGIB[2]—and (2) hyperglycemia-associated inflammatory activation and microvascular dysfunction, which may impair mucosal healing and clot stability[3].

These additions provide biological plausibility for the observed associations and better contextualize the study’s findings.

Changes in Manuscript:

The Discussion section was revised to include mechanistic explanations linking stress hyperglycemia with transfusion requirements and rebleeding risk.

**Reviewer Comment 2:**

**“Is it possible that stress hyperglycemia does not correlate with overall mortality because hyperglycemia triggers a more intensive treatment regiment? It would be helpful to comment on how local treatment algorithms are affected by blood glucose.”**

Response:

Thank you for this insightful comment. In our institution, blood glucose levels do not directly guide treatment escalation or triage decisions for patients with upper gastrointestinal bleeding. Management intensity is primarily driven by hemodynamic instability, ongoing bleeding, and end-organ dysfunction rather than glycemic status.

Due to IRB constraints, our dataset is fully de-identified and does not allow retrieval of detailed bedside management variables such as timing of vasopressor initiation. However, we performed additional exploratory analyses using all treatment-related variables available in the dataset, including transfusion, ICU admission, and interventions.

While patients with higher SHR were more likely to receive transfusion and ICU admission in the overall cohort, comparisons between survivors and non-survivors showed no significant differences in transfusion rates (87.8% vs. 100%, P = 0.603) or intervention rates (25.5% vs. 37.5%, P = 0.430). Although ICU admission was more frequent among non-survivors (62.5% vs. 15.1%, P = 0.004), this finding likely reflects clinical deterioration rather than hyperglycemia-triggered treatment escalation.

In contrast, non-survivors demonstrated markedly higher rates of acute complications, including AKI, ARF, and AMI, as well as a higher prevalence of chronic heart failure. These findings suggest that mortality in our cohort was driven predominantly by systemic decompensation and comorbidity burden rather than by differences in treatment intensity related to glycemic status.

Finally, the low overall mortality rate (2.3%) may have limited statistical power to detect a statistically independent association between SHR and mortality, raising the possibility of type II error.

Changes in Manuscript:

The Discussion section was revised to address whether stress hyperglycemia–related treatment escalation could have influenced the observed lack of association between SHR and overall mortality.

**Reviewer Comment 3:**

**“Baseline glycemic control is determined by A1c, which is perfectly reasonable, but it’s well known that anemia can affect A1c. This appears to be a confounder with the relationship between glycemic control, UGIB, and rebleeding. Please consider commenting on this. ”**

Response:

Thank you for raising this important point. HbA1c can indeed be affected by anemia through alterations in erythrocyte lifespan. Chronic iron-deficiency anemia may prolong erythrocyte survival and falsely elevate HbA1c, whereas conditions that shorten erythrocyte lifespan—such as acute blood loss or transfusion—can lower HbA1c independent of glycemia[4]. These mechanisms may introduce some measurement variability in estimating baseline glycemia.

However, large population-based analyses indicate that hemoglobin concentration itself has only a minimal impact on HbA1c, accounting for an absolute difference of approximately 0.2% (2.2 mmol/mol) across the full spectrum of hemoglobin values, even between the lowest and highest strata[5]. This suggests that acute reductions in hemoglobin—such as those occurring in upper gastrointestinal bleeding—are unlikely to meaningfully distort HbA1c values or materially bias the estimation of SHR in this cohort.

Changes in Manuscript:

The Discussion section was revised to address the potential impact of anemia on HbA1c measurement and its implications for SHR estimation.

We thank the Academic Editor and Reviewer for the thoughtful and constructive comments, which have substantially improved the clarity and rigor of this manuscript. We hope that the revised version adequately addresses all concerns and is now suitable for publication.

**References**

1.	Nathan DM, Kuenen J, Borg R, Zheng H, Schoenfeld D, Heine RJ, et al. Translating the A1C assay into estimated average glucose values. Diabetes care. 2008;31(8):1473-8.

2.	Laine L, Jensen DM. Management of patients with ulcer bleeding. Official journal of the American College of Gastroenterology| ACG. 2012;107(3):345-60.

3.	Turina M, Fry DE, Polk Jr HC. Acute hyperglycemia and the innate immune system: clinical, cellular, and molecular aspects. Critical care medicine. 2005;33(7):1624-33.

4.	English E, Idris I, Smith G, Dhatariya K, Kilpatrick ES, John WG. The effect of anaemia and abnormalities of erythrocyte indices on HbA1c analysis: a systematic review. Diabetologia. 2015;58(7):1409-21.

5.	Ford ES, Cowie CC, Li C, Handelsman Y, Bloomgarden ZT. Iron‐deficiency anemia, non‐iron‐deficiency anemia and HbA1c among adults in the US. Journal of diabetes. 2011;3(1):67-73.
